# A Study on the Radiosensitivity of Radiation-Induced Lung Injury at the Acute Phase Based on Single-Cell Transcriptomics

**DOI:** 10.3389/fimmu.2022.941976

**Published:** 2022-07-27

**Authors:** Luyu Ma, Yumeng Ye, Hao Lu, Yuan Xing, Zhen Zhao, Cheng Quan, Zhaoqian Jia, Yiming Lu, Yang Li, Gangqiao Zhou

**Affiliations:** ^1^Beijing Institute of Radiation Medicine, Beijing, China; ^2^Department of Rehabilitation Medicine, Eighth Medical Center, Chinese People’s Liberation Army (PLA) General Hospital, Beijing, China; ^3^The First Affiliated Hospital of Hebei North University, Zhangjiakou, China; ^4^Department of Pharmacy, Academy of Life Sciences, Anhui Medical University, Hefei, China; ^5^Collaborative Innovation Center for Personalized Cancer Medicine, Center for Global Health, School of Public Health, Nanjing Medical University, Nanjing, China

**Keywords:** radiation-induced lung injury, single-cell RNA sequencing, acute inflammation of lung injury, intercellular communication networks, radiosensitivity

## Abstract

**Background and Aims:**

Radiation-induced lung injury (RILI) is the most common complication associated with chest tumors, such as lung and breast cancers, after radiotherapy; however, the pathogenic mechanisms are unclear. Single-cell RNA sequencing has laid the foundation for studying RILI at the cellular microenvironmental level. This study focused on changes during the acute pneumonitis stage of RILI at the cellular microenvironmental level and investigated the interactions between different cell types.

**Methods:**

An acute RILI model in mice and a single-cell transcriptional library were established. Intercellular communication networks were constructed to study the heterogeneity and intercellular interactions among different cell types.

**Results:**

A single-cell transcriptome map was established in a mouse model of acute lung injury. In total, 18,500 single-cell transcripts were generated, and 10 major cell types were identified. The heterogeneity and radiosensitivity of each cell type or subtype in the lung tissues during the acute stage were revealed. It was found that immune cells had higher radiosensitivity than stromal cells. Immune cells were highly heterogeneous in terms of radiosensitivity, while some immune cells had the characteristics of radiation resistance. Two groups of radiation-induced Cd8^+^Mki67^+^ T cells and Cd4^+^Cxcr6^+^ helper T cells were identified. The presence of these cells was verified using immunofluorescence. The ligand-receptor interactions were analyzed by constructing intercellular communication networks. These explained the origins of the cells and revealed that they had been recruited from endothelial cells to the inflammatory site.

**Conclusions:**

This study revealed the heterogeneity of *in vivo* radiosensitivity of different cell types in the lung at the initial stage post irradiation

## Introduction

Radiation therapy (RT) is considered an effective method for the treatment of thoracic tumors. The lungs are moderately sensitive to ionizing radiation, and their susceptibility to radiation injury affects the curative effect of radiotherapy in lung cancer (1). Radiation-induced lung injury (RILI) refers to the radiation treatment of the chest, which may result in varying degrees of damage owing to a dose above the threshold that induces biological effects. Based on the different phases of injury, lung irradiation injury is typically divided into two stages. The first stage is early radioactive pneumonia, which occurs within hours to weeks of the acute inflammatory phase. The later stage is chronic radioactive pulmonary fibrosis, including tissue fibrosis, necrosis, and vascular injury, which is observed months to years after radiotherapy (2, 3). If a patient has RILI, not only will the quality of life and postoperative recovery be impacted adversely, but the rate of local tumor control will also be difficult to increase (1, 4). The overall effects of current clinical treatments remain poor, and there is a lack of effective therapeutic intervention methods for RILI. Therefore, it is of great significance to study the mechanisms of RILI.

In recent years, RILI has become a crucial topic in research, including its development as a complex process in which multiple factors interact with each other (1). After lung irradiation, the development of RILI begins with the production of large amounts of reactive oxygen species and nitrogen, which lead to DNA strand breaks and apoptosis. Next, alveolar epithelial cells and macrophages are damaged, releasing pro-inflammatory mediators and inflammatory cells, such as macrophages, eosinophils, and plasma cells. These cells are recruited and infiltrated into the affected area. In parallel, leukocytes or lymphocytes around the site of injury proliferate and produce cytokines and chemokines, leading to the proliferation of fibroblasts and a persistent inflammatory state. Accompanied by the activation of the immune system, this phase develops into an acute inflammatory phase (pneumonia) in which different lymphocyte subpopulations have heterogeneous functions and play distinct roles. For instance, Th1, Th17, and potential innate lymphoid cells can promote an inflammatory response, but Tregs are a group of cells that can control harmful and excessive pro-inflammatory responses (3). In such cases, lymphocytes are stimulated to produce lymphokines in contact with antigens to activate macrophages. In turn, cytokines produced by activated macrophages stimulate lymphocytes, which lay the foundation for a sustained inflammatory response (5).

At the late stage, the disease progresses slowly. Collagen deposition and fiber increase, while pulmonary fibrosis develops. This process involves a series of cellular and molecular changes due to cell death which secretes large amounts of inflammatory cytokines, chemokines, and growth factors. These attract more inflammatory cell aggregation. Continuous pneumonia peaks at the early stage and eventually develops into late irreversible lung fibrosis (1, 2).

It is unknown whether the radiosensitive cell population initiates the pathogenic process in the lungs. The development of RILI is the result of complex functional changes in the cellular microenvironment of the lungs, such as endothelial cells, macrophages, and other resident or recruited cells. Fortunately, single-cell RNA sequencing (scRNA-seq) analyses can provide a way to dissect these cells in a highly complex cellular microenvironment and study transcriptomic heterogeneity within specific cell populations (6–8).

In order to describe the heterogeneity of *in vivo* radiosensitivity of different cell types in the lung at the initial stage post irradiation, we performed scRNA-seq on all cells isolated from two strains of mice after radiation exposure. We then generated 18,500 single-cell transcripts and revealed the heterogeneity among lung tissue cells, especially the internal heterogeneity of immune cells. We found two groups of radiation-induced cells, *Cd8*^+^*Mki67*^+^ T cells and *Cd4*^+^*Cxcr6*^+^ helper T cells and verified the existence of these cells through immunofluorescence experiments. Finally, by constructing intercellular communication networks to analyze ligand-receptor interactions, we explained the origins of these cells and revealed that they had been recruited by endothelial cells to the inflammation site. Based on scRNA-seq, we discovered changes in the lung microenvironment at the initial stage of RILI and explored the interactions between multiple distinct immune cells and stromal cells when radiation-induced early inflammation occurred. Our findings will contribute to a more complete understanding of the mechanisms of RILI during the acute inflammatory phase.

## Materials and Methods

### Ethics Statement

The animal experiments conducted in this study were approved by the Institutional Animal Care and Use Committee of the Laboratory Animal Center. The experiments were conducted in accordance with the National Institutes of Health Guide for the Care and Use of Laboratory Animals.

### Experimental Animals and Groups

Sixteen male C57BL/6N and C3H/HeN mice (weight: 20 ± 2 g) were purchased from Beijing Vital River Laboratory Animal Technology Co. Ltd. (Beijing, China). All of the mice were maintained in a specific pathogen-free environment at a constant temperature of 22 ± 1°C, relative humidity of 60%, and a regular dark-light schedule (lights on from 7 a.m. to 7 p.m.) at the Experimental Animal Center of the Beijing Institute of Radiation Medicine, China. Sixteen mice were used in this study, including two random mice (one C57BL/6N and one C3H/HeN mice) receiving thoracic irradiation and two random mice (one C57BL/6N and one C3H/HeN mice) as negative controls. Lung samples were collected and used for scRNA-seq on day 1 post irradiation (n = 1 for each group). Twelve mice were used for the immunofluorescence (IF) analysis (n = 3 for each group).

### Irradiation

The mice were anesthetized with an intraperitoneal injection of 0.5% pentobarbital (43 mg/kg body weight). They were then exposed to a single dose of 20 gray (Gy) at a dose rate of 153.56 cGy/min. Irradiation was performed on the thoraces of the mice (from the clavicle to the lower edge of the costal arch with lead bricks to cover the other parts of the mice) using a cobalt-60 (^60^Co) γ-ray irradiator to induce lung injury *in vivo*. The mice were sacrificed 1 day after irradiation, and the lungs were collected for experiments.

### IF and Antibodies

For the IF analysis, mouse lung tissues were embedded in optimum cutting temperature compound and cut into 20 µm-thick sections using a freezing microtome (CM1950, Leica, Germany). The sections were fixed in 4% paraformaldehyde for 15 min at 4°C. They were blocked in IF buffer (10% normal goat serum (NGS), 1% bovine serum albumin (BSA), 0.3 M glycine, and 0.1% Tween 20) for 90 min at room temperature (15 - 25°C). The sections were then stained with primary antibodies (mouse-anti-CXCR6 1:200 and rabbit-anti-CD4 1:200; mouse-anti-CD8 1:400 and rabbit-anti-Ki67 1:500) diluted in 10% NGS IF buffer for 10 h at 4°C. The appropriate secondary antibodies, including goat anti-mouse Alexa Fluor 488 and goat anti-rabbit Alexa Fluor 594, were used to counterstain the sections for 90 min at room temperature. DAPI was used for nuclear staining, and the sections were mounted using Vectashield (H-1200-10, Vectorlabs, USA). The sections were observed with a fluorescence microscope (ECLIPSE CI-L, Nikon), and images were collected using NikonMosaic 1.6 software. The following experimental antibodies were used: mouse CXCR6 antibody (H00010663-B01, Annova, USA), rabbit KI67 antibody (9129, Cell Signaling Technology, USA), rabbit CD4 antibody (YT0762, Immunoway, USA), mouse CD8 antibody (66868-1-lg, Proteintech, China), goat pAb to mouse IgG (Alexa Fluor 488, ab150113, Abcam, USA), and goat pAb to rabbit IgG (Alexa Fluor 594, ab150080, Abcam, USA). The percentage of positive cells was used for a quantitative analysis. In brief, five visual fields were randomly selected, and a 40x objective lens was used for observation. The total numbers of DAPI stains and positive signals were counted. The percentage of positive cells was calculated as the number of positive cells divided by the total number of DAPI stains.

### Single-Cell Sequencing Library Construction

A single-cell suspension was obtained by first removing whole lung tissues from healthy mice and day 1 post irradiation mice. The tissues were cleaned with cold phosphate-buffered saline, cut into small pieces with lengths of approximately 2 mm, and separated according to the manufacturer’s instructions using the Lung Dissociation Kit (mouse) and GentleMACS Dissociator (Miltenyi Biotec, Germany). Next, the lung tissue mixtures were filtered through a 40-µm cell sieve on ice, centrifuged at 4°C, and washed twice with regular medium to remove incompletely digested fragments and tissues. There were approximately 5,000 single cells in each sample. The transcriptome sequencing analysis was processed on the 10x Genomics platform according to the manufacturer’s instructions. The Illumina HiSeq 3000 was used for deep sequencing.

### Preprocessing and Analysis of scRNA-seq Data

The 10x Genomics Cell Ranger software (version 2.2.0) was used to demultiplex the raw files into FASTQ files, extract barcodes, and align the reads from droplet-based scRNA-seq to the reference mouse genome (mm10, NCBI Build 38). This generated a gene expression matrix (unique molecular identifier [UMI] counts per gene per cell) for each sample. We then used standard Seurat (version 2.3.4) R toolkits (9) to integrate the output expression matrices collected from the four samples into the downstream analyses. By analyzing the datasets with the Seurat functions *NormalizeData*, *FindVariableGenes*, *ScaleData*, *RunMultiCCA*, *and AlignSubspace*, we removed the batch effect and carried out data normalization, scaling, downstream dimensionality reduction, clustering, and differential expression. Apart from running with default parameters, genes expressed in less than three cells and cells with less than 500 or over 8,000 expressed genes and over 15% UMIs derived from the mitochondrial genome were excluded at the quality control (QC) step. Consequently, 18,500 cells with a mean of 1,698 detected genes per cell were included in the following analysis. For all 18,500 cells, we identified clusters with a resolution number of 2. With the *FindClusters* function, we identified 31 clusters of different cell subtypes. The clustering results were visualized using a t-distributed stochastic neighbor embedding (t-SNE) dimensionality method.

### Identification of Marker and Differentially Expressed Genes

We identified the marker genes of each cell cluster using the Seurat function *FindMarkers* for significantly highly expressed marker genes. We filtered the results with an average log-transformed fold-change (logFC) > 1 and an adjusted *P*-value < 0.01. Next, we contrasted the results with known classic marker genes to distinguish each cell type. As a result, the whole data were divided into immune cells (macrophages, monocytes, granulocytes, T and natural killer [NK] cells, B cells, and dendritic cells [DCs]) and stromal cells (fibroblasts and endothelial, epithelial, and smooth muscle cells) according to the known markers: *Cd44*, *Lyz2*, *C1qa* (macrophages), *Ly6c2*, *Ms4a6c*, *Csf1r*, *F13a1* (monocytes), *Mpo* (granulocytes), *Cd3g*, *Gzma*, *Nkg7*, *Klre1* (T and NK cells), *Cd79a*, *Cd79b*, *Ighm*, *Igkc* (B cells), *Ccl17*, *H2-Eb1*, *Cd209a*, *Cd74* (DCs), *Cldn5*, *Kdr*, *Cdh5*, *Pecam1*, *Eng* (endothelial cells), *Dcn*, *Gsn*, *Col1a2*, *Col3a1*, *Col1a1*, *Mgp*, *Gpx3* (fibroblasts), *Acta2*, *Myl9*, *Mustn1* (smooth muscle cells), *Krt18*, *Krt8*, *Foxj1*, and *Sntn* (epithelial cells). Furthermore, we compared the differentially expressed genes within T cell populations and categorized 3,617 T cells into two clusters with a resolution parameter of 0.3.

### Gene Set Variation Analysis

We performed a gene set variation analysis (GSVA) with the GSVA (10) package (version 1.32.0) to estimate the pathway activation status of each cluster. First, we downloaded the 50 hallmark gene sets (version 6.2) representing unique biological processes from the Molecular Signatures Database (MSigDB) (11). We then converted the genes in each set to orthologous genes in mice using g:Profiler (12). With the default mode of the gsva function of GSVA and the *lmFit* function of the limma package (version 3.40.6), we detected the differentially enriched pathways in the pathway enrichment matrices of the gene datasets.

### Construction of Intercellular Communication Networks

The construction of intercellular communication networks was based on specific interactions between ligands and receptors. We collected 1,169 literature-supported and manually-curated ligand-receptor (L-R) interactions from the Fantom5 and CellphoneDB databases (13, 14). A Mann-Whitney U test was used to screen out the ligands and receptors that were significantly overexpressed in each cell cluster. The significance threshold was set at *P* < 0.05. When a corresponding ligand and receptor were highly expressed in two cell clusters, the L-R pair was called the “significant interaction pair”. The interaction intensity was defined as the sum of the upregulated rates of the ligand and receptor in the respective clusters. In addition, the total cellular communication strength between two cell clusters was defined as the number of significant L-R interaction pairs between them. These principles were used to construct intercellular communication networks in each cell subpopulation.

### Statistical Analysis

R (version 3.6.0) was used to perform all statistical analyses. We applied the chi-square test to analyze the distribution of the different cells and two strains before and after irradiation. The immunofluorescence staining data were analyzed using an unpaired t-test. We corrected the *P*-values for multiple tests using the Bonferroni correction method and concluded that an adjusted *P-*value < 0.05 was statistically significant.

## Results

### Single-Cell Sequencing in Healthy and Irradiation-Injured Mouse Lungs

**Figure 1:**
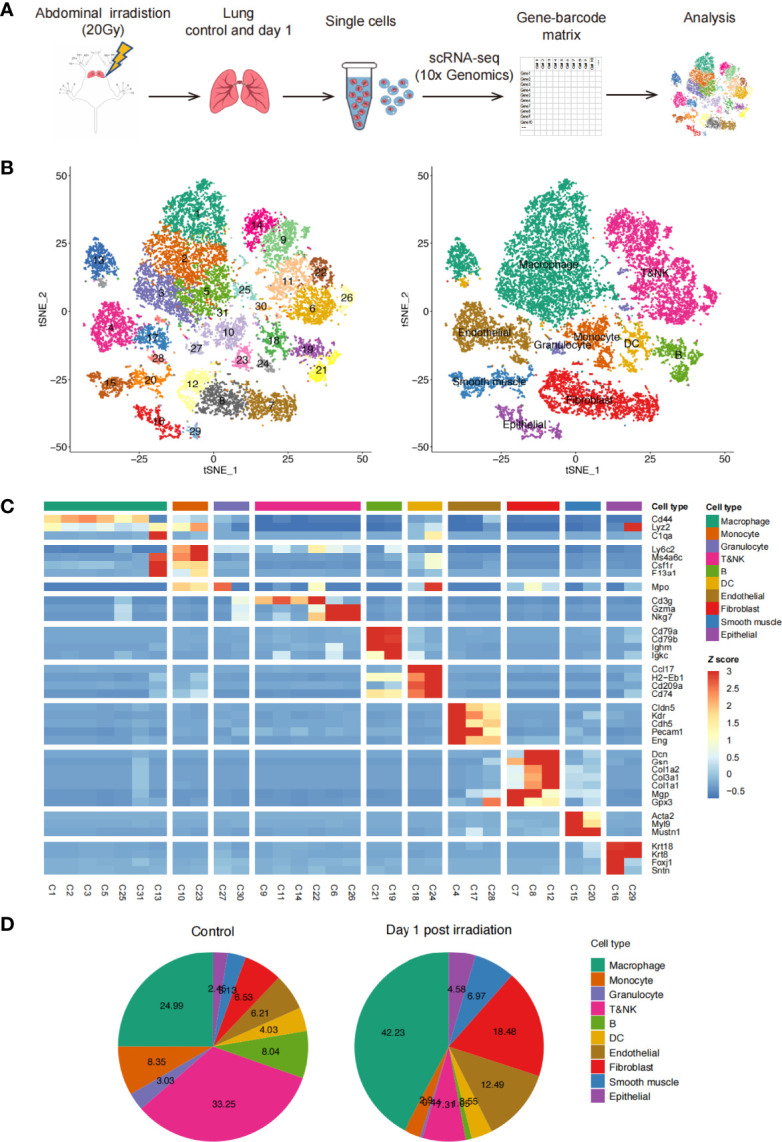
Profile of irradiation-induced lung injury (RILI) using scRNA-seq. **(A)** Schematic diagram of the experimental design and single-cell RNA sequencing (scRNA-seq) analysis. **(B)** t-SNE plot of 18,500 cells, color-coded by the associated clusters of Seurat cluster identities (left) and cell type annotations of samples (right). **(C)** Heatmap of cell type-specific and classical marker genes across clusters, measured as the z-score normalized mean expression. **(D)** Pie charts of cell type fractions before and after radiation among all samples, colored by cell type.

C57BL/6N and C3H/HeN mice were exposed to 20 Gy of thoracic irradiation using a ^60^Co irradiator to induce acute lung injury. We then used a droplet-based scRNA-seq approach to analyze the transcriptomes of single cells from the unexposed healthy lung tissues (n = 2) and irradiation-exposed lung tissues on day 1 (n = 2) (**Figure 1A**). After removing low-quality cells, the transcriptomes of 18,500 single cells, with an average of 1,698 genes and 5,032 unique transcripts per cell, were retained for further analysis (**Figure 1B**). By applying a canonical correlation analysis (CCA)-based batch correction approach, we merged the transcriptomes of single cells from all samples to generate a global map of the cellular microenvironments of healthy and injured lungs. By using the shared nearest neighbor (SNN) graph-based clustering of single cells, we identified 31 cell clusters (subtypes), which were visualized on the t-SNE dimensional reduction map (**Figure 1B**).

By using classic marker genes (15–19), the cell clusters in our dataset were readily identified as known cell types: lung endothelial cells, epithelial cells, fibroblasts, smooth muscle cells, myeloid cells (macrophages, monocytes, and granulocytes), T cells, NK cells, B cells, and DCs. Additionally, most of the major cell types were composed of multiple subtypes (**Figures 1C** and **S1A**). When comparing the percentages of each cell type in the lung tissues before and after irradiation, we found an increase in macrophages and stromal cells after irradiation. Conversely, the percentages of B, T, and NK cells decreased significantly (**Figure 1D**). We also compared the percentage of each cell type between C57BL/6N and C3H/HeN mice and found similar proportions between the two strains (**Figure S1B**). Overall, this analysis revealed the presence of a complex cellular ecosystem in healthy and irradiation-injured lungs containing 10 major cell types and 31 cell subtypes.

### Heterogeneity of *In Vivo* Radiosensitivity in Lung Multicellular Ecosystems

**Figure 2:**
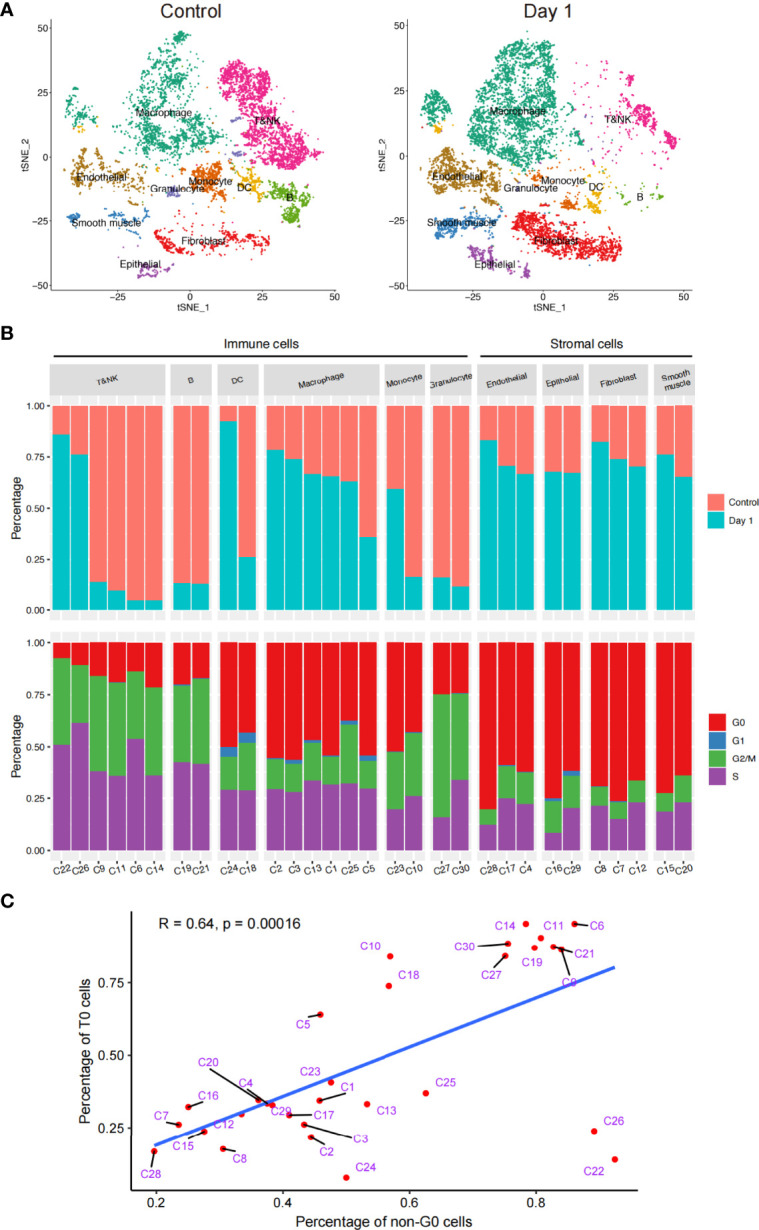
Schematic of the in vivo radiosensitivity of different cell subtypes in the lungs. **(A)** t-SNE plot of the 18,500 cells, colored by the cell distributions before radiation (left) and after radiation (right). **(B)** Bar plot (top) showing the fraction of cells before and after radiation in each cluster. Bar plot (bottom) showing the fraction of cells during the cell cycle. Only clusters that had > 50 cells were shown; therefore, cluster 31 has been discarded here. **(C)** Dot plot of the linear correlation between the ratio of unirradiated cells and G0 phase cells of each cluster, not including cells of cluster C31.

Given the changes in the cellular components of the lungs before and 1d after irradiation, we explored the *in vivo* radiosensitivity of multifarious cell subtypes (**Figure 2A**). We compared the percentages of each cell cluster between the healthy and post-irradiated lung samples to quantify the radiosensitivity of each cluster. Our data indicated considerable differences in the radiosensitivity of stromal cells and most immune cell populations. In contrast to the stromal cells, most of the immune cell subtypes had significantly lower frequencies after irradiation than the healthy lung samples, indicating that the immune cells were highly radiosensitive. This is consistent with previous studies (20). However, different immune cell populations showed different radiosensitivity, such as myeloid cells (macrophages, monocytes, and granulocytes) and B cells, which exhibited highly radiosensitive phenotypes. We found that the immune cells exhibited heterogeneous levels of radiosensitivity across their respective subtypes. Our data revealed that a few DC, T, and NK cell subtypes (e.g., clusters C22, C24, and C26) were enriched after irradiation, indicating they were more likely to be radiation-induced compared to other subtypes. In parallel, we performed a cell cycle analysis of the subpopulations of immune and stromal cells. The results showed that the T, NK, B cells, and DCs were abundant in the S phase, suggesting that they may have been in the DNA replication and synthesis stages. In contrast, each stromal cell cluster had a large number of cells in the G0 phase, indicating that they were in a stable state (**Figure 2B**).

The relationship between the cell cycle and radiosensitivity garnered our attention. A correlation analysis revealed a significant negative linear correlation between the proportion of unirradiated cells in all cells of each cluster and the proportion of cells in the G0 phase out of all four cell cycle stages. These results suggested that if cell division was actively taking place, then the radiosensitivity of the cell would be lower (**Figure 2C**). In addition, although the C22 and C26 cells had strong radiation resistance, cell division was taking place actively; therefore, we carried out further analyses.

We sought to study radiosensitivity at the level of the activated cellular pathways. To characterize the function of each subpopulation in greater detail, we used GSVA to compare the functional pathway activities of different cell clusters, focusing on the radiosensitive and radioresistant cell types in our dataset including myeloid, T, and NK cells (**Figure S1C**). We found that alveolar macrophages (clusters C1, C2, C3, C5, C25, and C31) were significantly upregulated in many signaling pathways, including the oxidative phosphorylation, tumor necrosis factor (TNF)-α signaling *via* nuclear transcription factor kappa-B (NF-κB), and PI3K/Akt/mTOR signaling pathways. Interstitial macrophages (cluster C13) were highly expressed in the p53 and interferon-γ response pathways. Monocytes (clusters C10 and C23) were highly expressed in the interferon-α and interferon-γ response pathways. Granulocytes (clusters C27 and C30) were highly expressed not only in the interferon-α and interferon-γ signaling pathways, but also in the spermatogenesis and KRAS signal response pathways. These results are consistent with the conclusions of previous studies. For example, at the early stage of acute lung injury, NF-κB was found to be activated by bacterial endotoxins, participated in transcription, and produced pro-inflammatory cytokines such as TNF-α, interleukin (IL)-1β, and IL-6. It has further been shown that TNF-α signaling *via* the NF-κB pathway plays an important role in acute lung injury by producing many pro-inflammatory cytokines (21). Hence, our analysis suggested that the activation of the aforementioned pathways may be related to the process of acute lung injury.

The GSVA results of the T and NK cell pathway activities indicated that the T cell subpopulations C9 and C14 were significantly upregulated in only a few pathways, such as the Wnt/β-catenin and bile acid metabolism signaling pathways (**Figure S1D**). However, the T cell subpopulations C11 and C22 were significantly upregulated in many pathways, such as the DNA repair, interferon α-signaling, and G2/M checkpoint pathways. The NK cell subpopulations (clusters C6 and C26) were significantly upregulated in the apical binding and complement signaling pathways. These results suggested that the activation of many pathways may be related to the process of acute lung injury. In line with previous studies, a recent study (22) proved that miRNA1246 could mediate the inflammatory and apoptotic effects at the acute lung injury stage by inhibiting Wnt/β-catenin and activating NF-κB signaling pathways.

**Figure 3:**
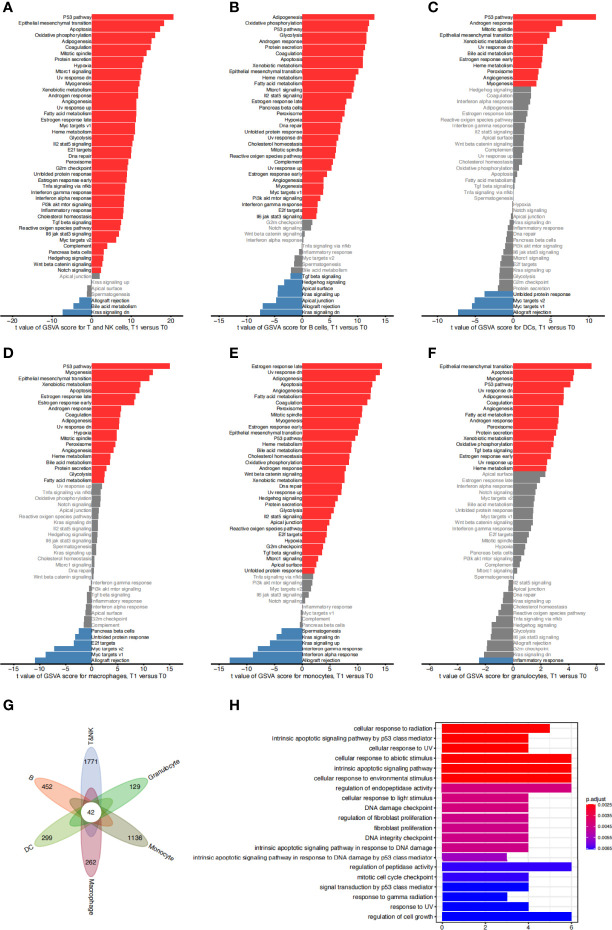
Functional characteristics of sensitive cell types. **(A)** Difference of hallmark pathway activities between sensitive T&NK clusters (C6, C9, C11, C14) from the control group and 1 day post irradiation. Shown are t values calculated in a linear modal comparing the pathway scores estimated by gene set variation analysis (GSVA) between cells from the two groups. **(B–F)** The same as **(A)** for sensitive B clusters (C19, C21) **(B)**, DC clusters (C18) **(C)**, macrophage clusters (C5) **(D)**, monocyte clusters (C10) **(E)** and granulocyte clusters (C27, C30) **(F)**. **(G)** Venn diagram showing the intersection of significantly upregulated genes of sensitive cells of each cell type from 1 day post irradiation compared to that from the control group. **(H)** The top 20 significantly enriched gene ontology (GO) terms of 42 common upregulated genes for sensitive T&NK, B, DC, macrophage, monocyte and granulocyte clusters.

We next sought to identify the signaling cascades triggered in various cell types to confront irradiation-induced apoptosis. We focused on the radiosensitive clusters for different immune cell types, and compared the GSVA pathway activities in cells from 1 day post irradiation to those from the control group. Results showed that a number of pathways were consistently upregulated in the surviving cells from various immune cell types compared to the non-irradiated cells, including the P53 pathway, apoptosis, and ultraviolet response down pathways (**Figures 3A–F**). We next examined the differentially expressed genes in these surviving cells and identified 42 significantly upregulated genes which were shared by all the radiation-sensitive immune cells (**Figure 3G** and **Supplementary Table S1**). Gene Ontology (GO) term annotation showed that these upregulated genes were enriched in biological processes including cellular response to radiation and abiotic stimulus, intrinsic apoptotic signaling pathway, and DNA damage related pathways (**Figure 3H**). These results revealed the common pathways and genes that may be involved in the radiation-induced responses in different radiosensitive immune cells.

We further detected the *in vivo* radiosensitivity of various cell subtypes in the two mouse strains, C57BL/6N and C3H/HeN (**Figure S2A**). We also compared the proportions of all cell subtypes in each strain before and after irradiation to identify the differences in radiosensitivity (**Figure S2B**). We found that there were radiosensitivity trends in the immune and stromal cells in the two mouse strains. For example, the immune cells exhibited higher radiosensitivity. However, there was no significant difference in the radiosensitivity between the two mouse strains.

### Changes in T Cell Subtypes in Healthy Lungs and RILI

Following the above analysis, we found that the radiosensitivity of lung tissues varied greatly among all cell types. In particular, it seemed that different subpopulations of T and NK cells had different radiosensitivity and levels of expression in the functional pathways (**Figures 2B** and **S1D**).

We identified marker genes (23–25) for T and NK cells as specific cell subtypes. Cells in clusters C9 and C14 expressed naïve T cell signatures *(Tcf7*, *Sell*, *Lef1*, and *Ccr7*) highly, suggesting that they were naïve T cells. Cells in clusters C6 and C26 expressed the signatures of NK or NK T cells (*Klrb1c* and *Klrd1*) highly, suggesting that they were NK or NK T cells. Cells in cluster C11 expressed high levels of regulatory T cell (Treg) signatures (*Il2ra*, *Foxp3*, and *Ikzf2*), suggesting that they were a set of Tregs. Cluster C22 was enriched with T cells that expressed *Cd8b1* highly, which is a signature of recently reported *Cd8*^+^ T cells. We found that the cells in cluster C22 also expressed high levels of classic T cell receptor genes (*Trac*).

**Figure 4:**
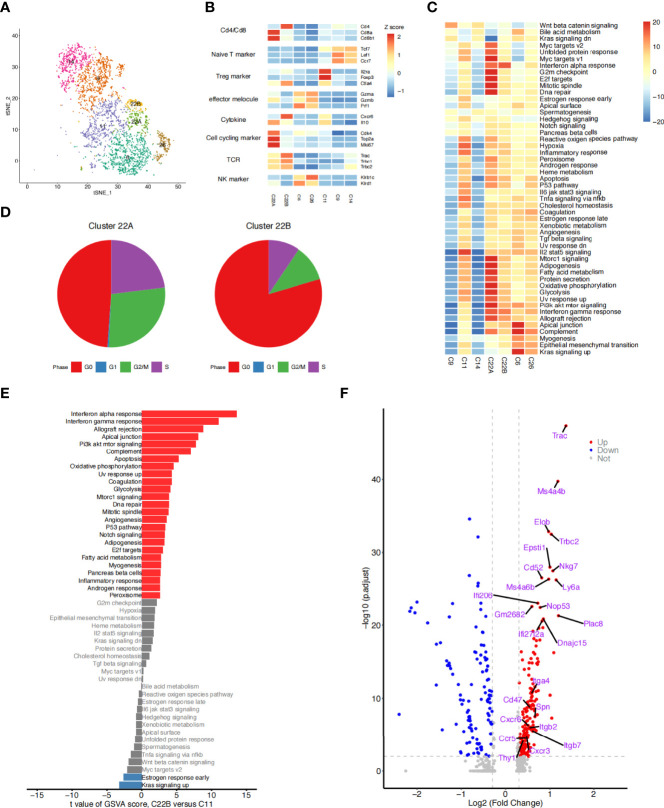
Detailed characterization of T/NK cells. **(A)** t-SNE plot of 3,617 T and NK cells, color-coded by the re-clustered cell subtypes. **(B)** Heatmap of the average expression of selected function-related marker genes in seven T and NK cell clusters. **(C)** Heatmap of the differences in pathway activities between seven clusters of T and NK cells. Data shown are t values scored per cell from a linear model between every two groups by gene set variation analysis (GSVA). **(D)** Pie plots showing the fraction of the cells in clusters C22A and C22B during the cell cycle. **(E)** Bar plots showing differences in the hallmark pathway activities of T cells of two groups: clusters C22B and C11. Data shown are t values scored per cell from a linear model between the two groups by GSVA. The red columns represent the significantly upregulated functional pathways in C22B cells compared with C11 cells (P ≤ 0.05, t > 0); the blue columns represent the downregulated functional pathways in C22B cells compared with C11 cells (P ≥ 0.05, t < 0); and the gray columns represent the functional pathways that were not significantly expressed in C22B cells compared with C11 cells (P ≥ 0.05). **(F)** Volcano diagram displaying the differentially expressed genes (T cells of C22B versus C11). Each red dot was considered a significantly upregulated gene with an adjusted P value < 0.01 and fold-change ≥ 0.3; each blue dot was considered a significantly downregulated gene with an adjusted P value < 0.01 and fold-change ≤ -0.3; and the grey dots were considered insignificant genes (*P* value > 0.01 and -0.3 < logFC < 0.3). The first 15 genes that were significantly upregulated and the 9 receptor genes that were previously screened are labeled.

A closer examination of the cells in cluster C22 showed two different subclusters (C22A and C22B) (**Figure 4A**). C22A cells expressed higher levels of *Cd8b1* and cell cycle-related genes (*Mki67*). CD8 (Cd8b1) (26, 27) is a transmembrane glycoprotein that is predominantly expressed on the surface of cytotoxic T cells. It plays a role in T cell development and the activation of mature T cells. Mki67 (Ki67) (28) is a nuclear non-histone protein that is universally expressed in proliferating cells and absent in quiescent cells. These results indicated that C22A cells were likely to be *Cd8*^+^ T cells in an actively proliferating state. In contrast, cells in C22B expressed not only Treg classical marker genes (*Il2ra*, *Foxp3*, and *Ikzf2*), but also a higher level of *Cd4*^+^*Cxcr6*^+^ out of the helper T cell (Th) genes (29). CXCR6 is the receptor for the C-X-X chemokine CXCL16, which is expressed in lymphoid tissues and activated T cell membranes (30). CD4 encodes a membrane glycoprotein of T lymphocytes that interacts with major histocompatibility complex class II antigens (24). Therefore, we hypothesized that this proportion of Tregs may have been *Cd4*^+^*Cxcr6*^+^ Th cells (**Figure 4B**). Finally, we reclustered the T and NK cells into five subtypes: *Cd8*^+^*Mki67*^+^T cells (C22A), *Cd4*^+^*Cxcr6*^+^Th cells (C22B), NK cells (C6 and C26), Tregs (C11), and naïve T cells (C9 and C14). t-SNE plotting revealed that the chemokine *Cxcr6* was abundant among the Th cells and Tregs (C22B and C11), which confirmed our assumption (**Figures 4A, B**).

**Figure 5:**
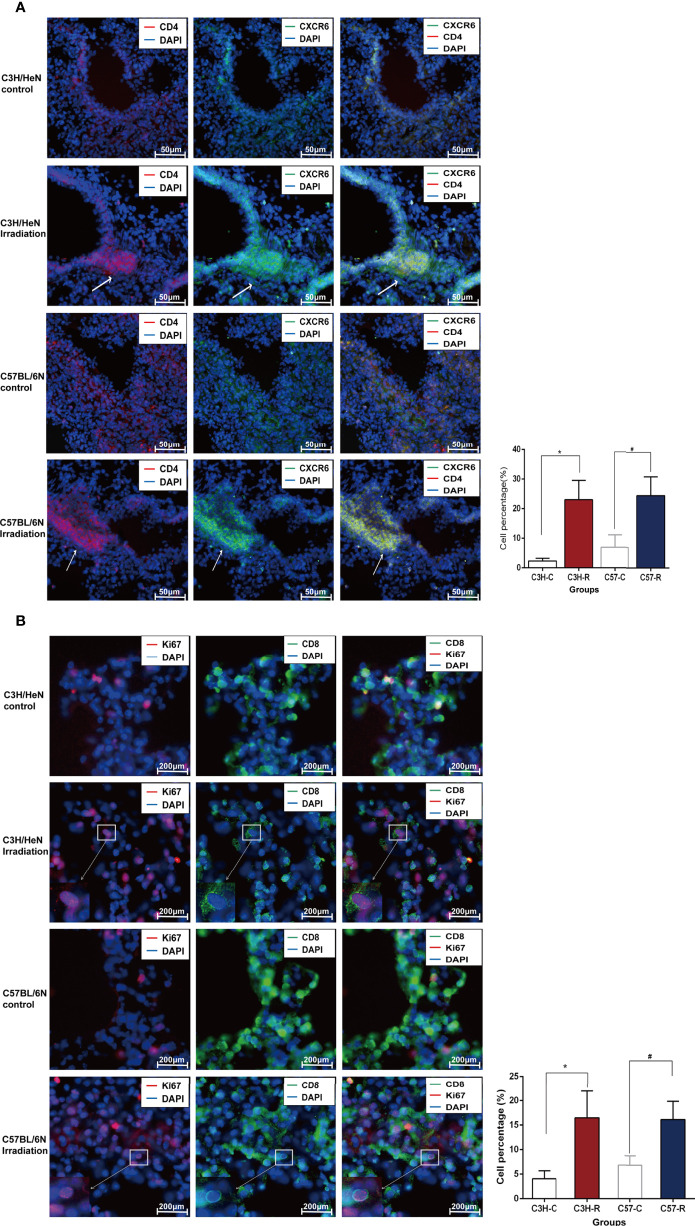
Immunofluorescence data confirmed the existence of the subset of T cells after radiation. **(A)** Immunofluorescence staining diagrams of genes Cd4 and Cxcr6 in lung tissue before and after radiation. **(B)** Immunofluorescence staining diagrams of genes Cd8 and Mki67 in lung tissue before and after radiation.

Given that the cells of clusters C22A and C22B were enriched after irradiation (**Figure 4A**), we speculated that these cells may have been recruited by other cytokines. We conducted immunofluorescence experiments to identify whether cells with high expressions of *Cd8*, *Mki67*, *Cd4*, *Ctla4*, and *Cxcr6* could be induced by irradiation. The immunofluorescence data confirmed the existence of this subset of T cells by the co-expression of *Cd8* and *Mki67*. Moreover, *Cd8*^+^*Mki67*^+^ T cells were scattered in the lungs on day 1 post irradiation, but only a few were found in the lungs of the control mice. There was no significant difference between the C57BL/6N and C3H/HeN mice (**Figure 5B**). The existence of this subset of T cells by the co-expression of *Cd4* and *Cxcr6* was also confirmed by immunofluorescence. Moreover, there were more *Cd4*^+^*Cxcr6*^+^ Th cells in the lungs of the irradiated mice, but only a few were found in the lungs of the control mice. There was no significant difference between C57BL/6N and C3H/HeN mice. Interestingly, *Cd4*^+^*Cxcr6*^+^ Th cells were specifically enriched in the blood vessels, suggesting that these activated T cells were recruited from the blood vessels to the lungs after irradiation (**Figure 5A**). After assessing additional samples by immunofluorescence, we further validated the aforementioned speculations. We concluded that the T cells of clusters C22A and C22B had been recruited after irradiation and that the *Cd4*^+^*Cxcr6*^+^ Th cells had been recruited from blood vessels to the lungs.

### Analysis of the Difference Between Two T Cell Subtypes in the Cell Microenvironment of Acute RILI

We compared the pathway activity levels of T and NK cell subsets, focusing on the cell cycle phase between clusters 22A and 22B (**Figures 4C, D**). The GSVA results were consistent with the cell cycle analysis in which C22A cells expressed higher levels of G2/M checkpoint and DNA repair pathways than C22B cells. The proportion of C22A cells at the G2/M and S stages was also significantly higher than that of C22B cells. As previously reported (31), irradiation-induced cell cycle checkpoint signaling pathways facilitate cell cycle stagnation, which boosts cell survival in response to irradiation.

Although the cells in clusters C22B and C11 expressed the classical marker genes (*Il2ra*, *Foxp3*, *Ctla4*, and *Ikzf2*) of Tregs highly, the Th cells in cluster C22B were specifically enriched after irradiation. In contrast, the Tregs in cluster C11 were enriched before irradiation (**Figure 4B**). Therefore, we aimed to identify the inter-individual differences between the two groups of cells by researching the functional pathways and gene levels.

Follow-up GSVA results showed that pathways were significantly up/downregulated in the whole cells of cluster C22B compared with those of C11. Interestingly, a large number of pathways were consistently upregulated in C22B (**Figure 4E**), including the interferon-α and interferon-γ response signaling pathways. It has long been recognized that interferons (32, 33) not only play pivotal roles in the DNA damage-induced inflammatory response among irradiated cells, but are also necessary parts of the innate cytokine response to viral infection; indeed, they have many other important functions in the immune system. We also analyzed the genes that were significantly upregulated in cluster C22B compared with those in C11, including various chemokines and integrins, such as *Cxcr6*, *Cxcr3*, *Itga4*, and *Itgb2* (**Figure 4F**). Based on the results, we suspected that the reason for the enrichment of C22B cells after irradiation may have been that radiation-induced interactions between ligands in other cells and highly expressed receptors (such as chemokine *Cxcr6*) in *Cd4*^+^*Cxcr6*^+^Th cells, which are recruited to inflammatory sites and play a role in suppressing inflammation.

### Intercellular Communication in the Cell Microenvironment of Acute RILI

In the cellular microenvironment, all types of cells obtain regulatory signals from upstream pathways, then quickly transmit signals and induce other cells to participate in the process. This forms complex intercellular communication networks (34–36). Different cells interact with each other through a variety of molecular forms, such as receptors, ligands, ions, integrins, junction proteins, and extracellular matrix proteins. Ligands such as chemokines, cytokines, hormones, growth factors, and neurotransmitters can mediate intercellular communication, or cell-cell communication (37).

Previous studies have reported that Tregs express a repertoire of inflammatory chemokine receptors (e.g., CCR2 and CXCR6) and cytokines (e.g., FOXP3 and CTLA4), suggesting that these cytokines may mediate the recruitment of Tregs at inflammatory sites by binding to their receptors on the Tregs (38). We hypothesized that the irradiation-induced inflammatory responses of stromal cells, such as endothelial cells, lead to the recruitment of *Cd4^+^Cxcr6^+^* Th cells. We investigated the origin of this group of cells and assessed the role of L-R pairs in mediating cellular interactions among clusters.

**Figure 6:**
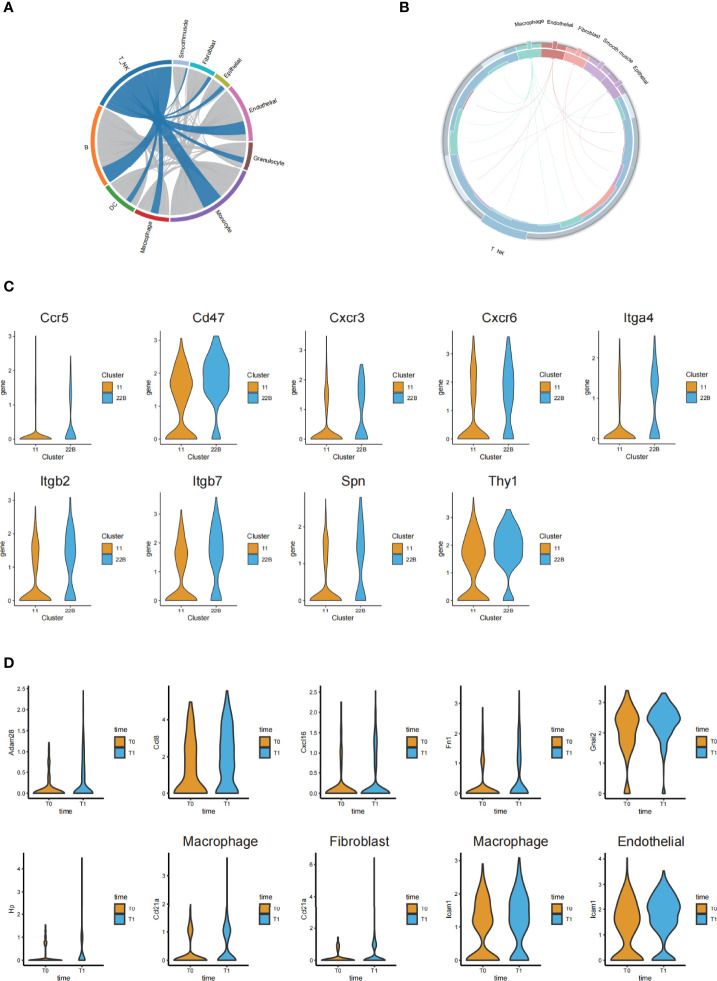
Ligand-receptor-based interaction between immune cells and stromal cells. **(A)** Circos diagram of the weighted numbers of ligand-receptor interaction pairs between paired interactions of the upregulated genes in different cell types (post-irradiation versus pre-irradiation). **(B)** Circos diagram showing the 17 upregulated ligand-receptor interactions between the T or NK cells and stromal cells. **(C)** Violin plots showing the significantly upregulated receptor genes from the cluster C22B and C11 of T cells. **(D)** Violin plots of the corresponding and significantly upregulated ligand genes from other cells after irradiation.

By comparing the changes in the differentially expressed genes and L-R pairs before and after irradiation, we constructed intercellular communication networks in post-irradiation lung tissue. When visualized through the Circos figure, we found 626 upregulated L-R interactions among all cell types. A large number of interactions between T or NK cells and monocytes were further observed (**Figure 6A**).

Although cellular interaction patterns varied greatly among the cell types, we focused on the clusters of T cells; in particular, we screened out the receptor genes with significantly upregulated and higher expression in cluster C22B than in C11 (**Figure 6C**). We found that C22B cells expressed nine receptors (including Ccr5, Cxcr3, Cxcr6, Itga4, Cd47, Itgb2, Itgb7, Spn and Thy1).

To analyze the cells that had L-R pairs that were significantly related to T and NK cells, we concentrated on the corresponding and significantly upregulated ligand genes of other cells (macrophages, fibroblasts, endothelial cells, epithelial cells, and smooth muscle cells) paired with the nine C22B-expressed receptors after irradiation (**Figure 6D**). We found that epithelial cells (cluster C16) expressed the significantly upregulated ligand Adam28, which was paired with the receptor Itga4 in T and NK cells. Macrophages (cluster C13) expressed the significantly upregulated ligand Ccl8, which was paired with the receptor Ccr5 in T and NK cells. Endothelial cells (cluster C4) expressed the significantly upregulated ligand Cxcl16, which was paired with the receptor Cxcr6 in T and NK cells. Macrophages (cluster C5) expressed the significantly upregulated ligand Fn1, which was paired with receptors Itgb7 and Itga4 in T and NK cells. The endothelial cells (clusters C4 and C17) expressed the significantly upregulated ligand Gnai2, which was paired with receptor Ccr5 in T and NK cells. The macrophages in clusters C5 and C25 expressed the significantly upregulated ligand Hp, which was paired with receptor Itgb2 in T and NK cells. The macrophages in clusters C2, C5, and C25, fibroblasts in clusters C7 and C8, smooth muscle cells in cluster C20, and epithelial cells in cluster C29 were all rich in the ligand Ccl21a, which was paired with the receptor Cxcr3 in T and NK cells. Both macrophages in cluster C5 and endothelial cells in cluster C4 expressed the significantly upregulated ligand Icam1, which was paired with receptors Itgb2 and Spn in T and NK cells. We hypothesized that these L-R pairs, which were significantly related to T and NK cells, may be closely related to acute lung injury.

Further investigation of 17 upregulated L-R interactions between the T and NK cells and other cells provided relevant evidence for our supposition (**Figure 6B**). The results of the intercellular communication networks suggested that there was a close relationship between the T and NK cell and endothelial cell subpopulations, which was consistent with the results of the immunofluorescence assay.

Together with previous studies (39), our analytical and experimental results indicated that activated endothelial cells expressed high levels of adhesion molecules such as Cxcl16, the Cxcr6 ligand, to recruit pro-inflammatory T cells to inflammatory sites. We also found two groups of T cell subpopulations (*Cd8*^+^*Mki67*^+^T cells and *Cd4*^+^*Cxcr6*^+^Th cells) that were enriched after irradiation and found that *Cd4*^+^*Cxcr6*^+^Th cells may be recruited to inflammatory sites due to the corresponding ligand (Cxcl16) secreted by endothelial cells. This could play a role in inhibiting inflammation.

## Discussion

RILI comprises all pulmonary toxicities and presents two main phases of the radiation response: acute radiation pneumonia and chronic radiation pulmonary fibrosis (40). It is unclear how changes in the cellular microenvironment and intercellular interactions affect the process of acute lung injury. Here, we combined single-cell transcriptome, gene set difference, cell cycle, and cell-cell interaction analyses to demonstrate the changes of the cellular microenvironment at the initial stage post irradiation

Radiation induces cell death through direct cytotoxicity. Cell sensitivity to radiation varies greatly under many conditions, such as cell type, degree of cell differentiation, proliferation ability, metabolic status, and the influence of the surrounding environment (41, 42). Generally, immune cells, such as lymphocytes, are considered radiosensitive cell types, which are composed of a variety of cell subtypes with biologically distinct properties. Consistent with previous studies (43), our data showed that immune cells were more sensitive to radiation than stromal cells; the activity of cell division increased; and the sensitivity of cells post-radiation decreased. In addition, by using single-cell sequencing technology, we not only developed a systematic map of the heterogeneity of the evolutionary lung microenvironment *in vivo* in both healthy and post-radiation states, but also revealed the cross-cell consistency of pathway activation among various cell types.

A large degree of variation in the radiosensitivity of different immune cell subtypes was found. For instance, some subtypes were sensitive, while other subtypes were insensitive (subsets C22 and C26 of T and NK cells, subset C24 of DC cells, respectively). When comparing the differentially expressed genes, we focused on the C22 cluster of T cells and reclustered them into two subtypes: C22A, the proliferated *CD8^+^Mki67*^+^T cells, and C22B, the *Cd4*^+^*Cxcr6*^+^Th cells. To corroborate these profiles, we considered it necessary to perform an immunofluorescence assay to verify that the aforementioned genes were enriched in the lung after radiation.

Next, we followed cues from an immunotherapy study (30) by Mee Sun Yoon et al., who demonstrated that cells expressing a soluble CXCL16 ligand could induce the immune cell expression of the CXCR6 receptor (*CD8*^+^ T cells, *CD4*^+^ T cells, and NK T cells) and chemotactic recruitment to inflammatory sites. In order to study the causes and origins of the high expression of *Cxcr6* in post-irradiation T cells, we validated the interactions between cells. Cell-cell interactions (44) play a key role in studying the origin of disease through L-R communication. Therefore, based on the known ligand and receptor pairs co-expressed in any two cell types, we constructed an intercellular communication network model. A systematic analysis including the gain of potential cell-cell interactions, obtaining L-R pairs related to the T and NK cells, and observing the T and NK cells closely related to the endothelial cells after irradiation was implemented. Collectively, the results of the immunofluorescence experiments suggested that the increased population of *Cd4*^+^*Cxcr6*^+^Th cells after radiation accumulated around the blood vessels. The computational analysis and experiments showed that activated endothelial cells expressed high levels of Cxcl16 as a ligand to recruit T cells expressing receptor Cxcr6 to inflammatory sites after irradiation.

By using scRNA-seq, we obtained a single-cell transcriptional map of healthy lung tissue and RILI at the initial stage post irradiation in mice to reveal the nature of single-cell subtypes. In addition, we identified the radiosensitivity and resistance of each cell subtype after radiation from several aspects. Moreover, we discovered radioresistant cell subpopulations and performed experiments to verify the existence and origin of these cells. Finally, through intercellular communication networks, we explained why these cells were recruited by other cell subpopulations. The discovery of radiosensitive cells could protect the immune response of sensitive cells and promote the recovery of tissues and organs. The increase in the number of radiation-resistant cells may point to a reparative treatment option that could be used in the early intervention of lung injury. These findings therefore provide potential novel targets and strategies for the development of immunotherapy in treating early-stage RILI.

## Data Availability Statement

The data presented in the study are deposited in the GEO repository. https://www.ncbi.nlm.nih.go v/geo/query/acc.cgi?acc=GSE206426. Accession number: GSE206426.

## Ethics Statement

The animal experiments conducted in this study were approved by the Institutional Animal Care and Use Committee of the Laboratory Animal Center. The experiments were conducted in accordance with the National Institutes of Health Guide for the Care and Use of Laboratory Animals.

## Author Contributions

GZ, YL, and YML supervised the study. LM, YY, and HL performed experiments and analyzed data. YX, ZZ, CQ, and ZJ participated in mouse modeling and dissection. LM, YY, and HL wrote the paper. All authors contributed to the article and approved the submitted version.

## Funding

This work was funded by the General Program (NO. 31771397 and NO. 81573251) of the Natural Science Foundation of China (www.nsfc.gov.cn), the Beijing Nova Program (NO. 20180059) and the Scientific Research Project (NO. BWS21J022 and NO. AWS21J003).

## Conflict of Interest

The authors declare that the research was conducted in the absence of any commercial or financial relationships that could be construed as a potential conflict of interest.

## Publisher’s Note

All claims expressed in this article are solely those of the authors and do not necessarily represent those of their affiliated organizations, or those of the publisher, the editors and the reviewers. Any product that may be evaluated in this article, or claim that may be made by its manufacturer, is not guaranteed or endorsed by the publisher.
