# Supplementary figures and images for: A Study on the Radiosensitivity of Radiation-Induced Lung Injury at the Acute Phase Based on Single-Cell Transcriptomics
Source: Front Immunol. 2022 Jul 27;13:941976. doi: 10.3389/fimmu.2022.941976 (PMC9364823; doi:10.3389/fimmu.2022.941976)

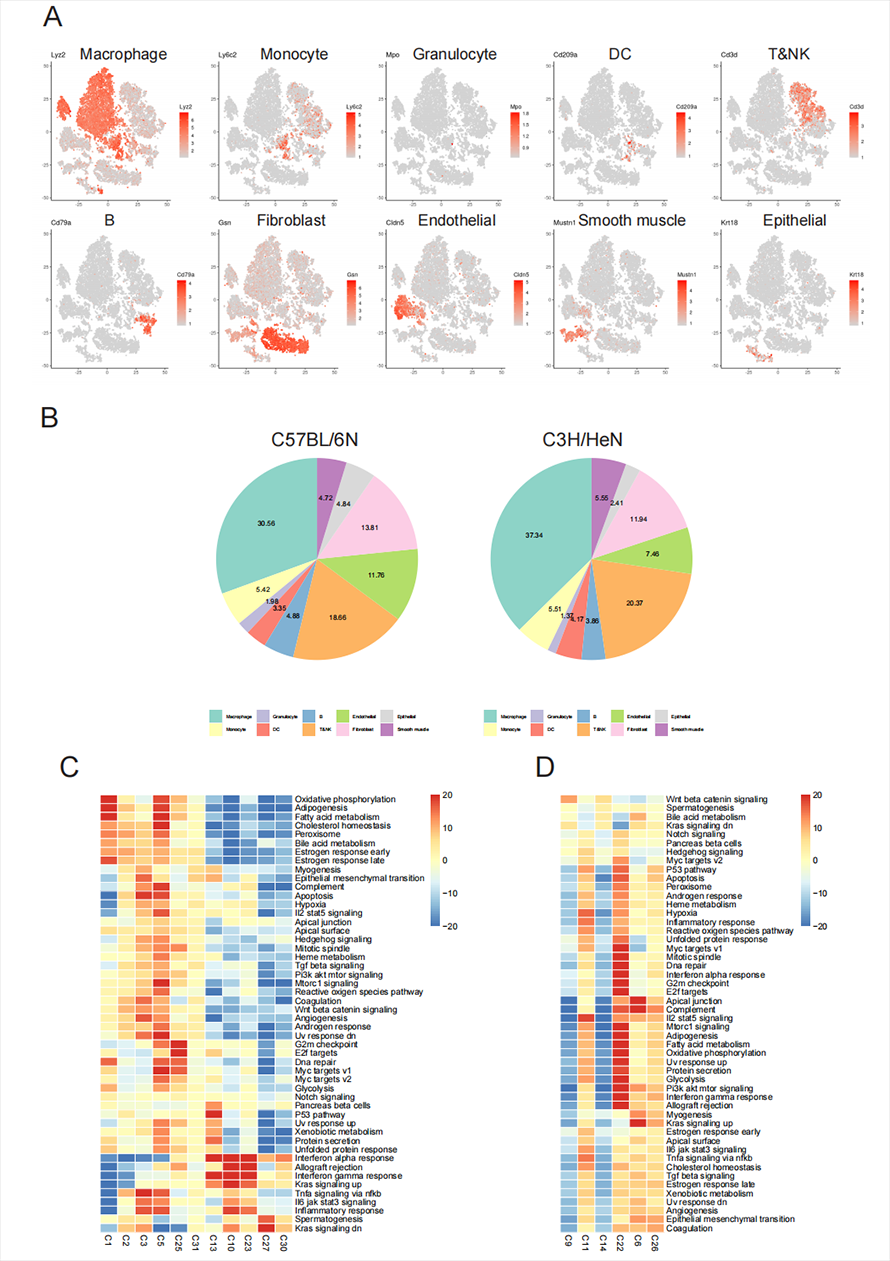

Supplement: Supplementary file 1 [file Image_1.tif]

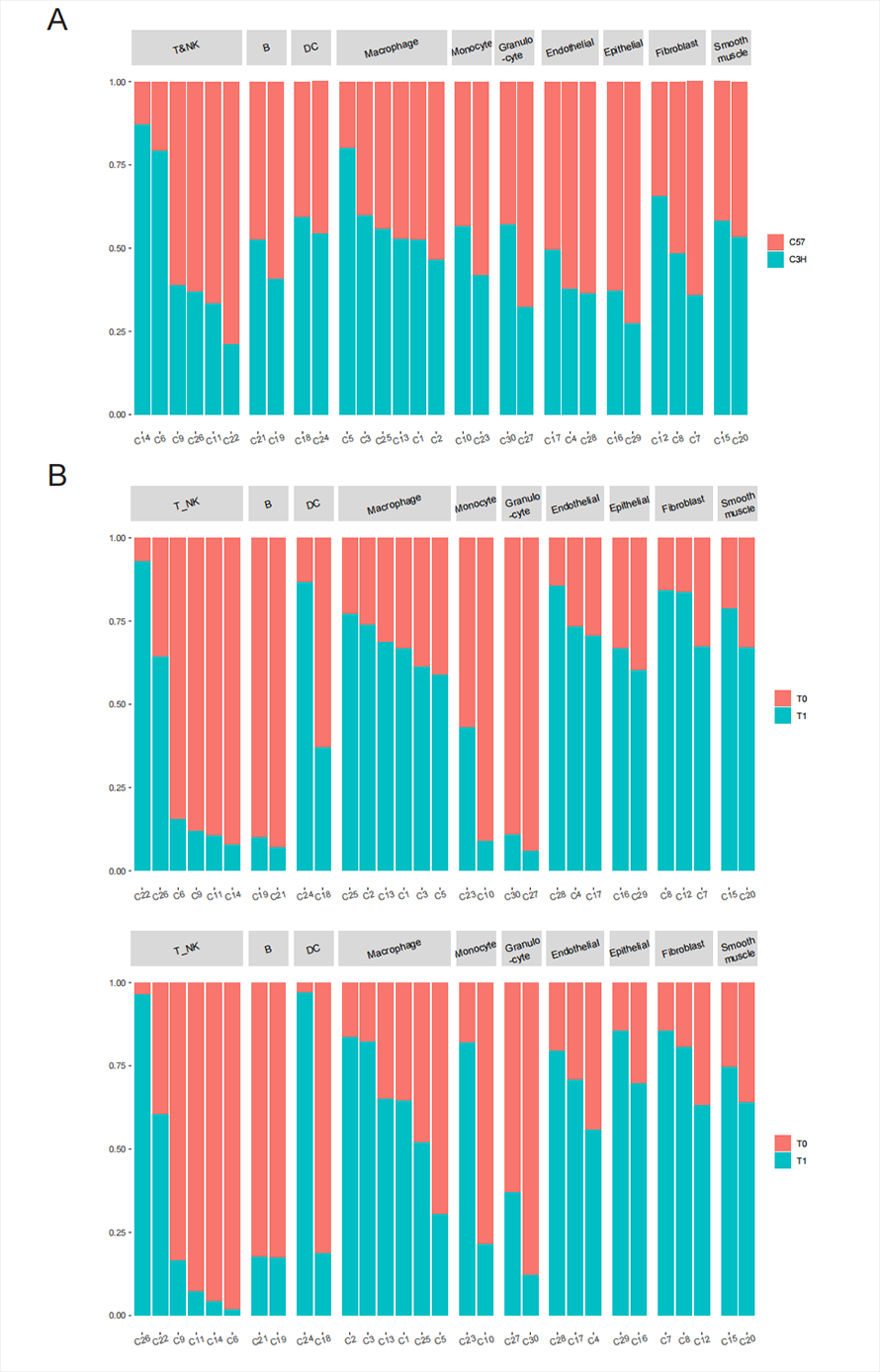

Supplement: Supplementary file 2 [file Image_2.tif]
